# Nexus Between Economic Efficiency, Healthcare, and Environmental Expenditures: A Perspective of BRI Countries

**DOI:** 10.3389/fpubh.2022.842070

**Published:** 2022-02-09

**Authors:** Zahid Hussain, Cuifen Miao, Zhihao Zhao, Yingxuan Wang

**Affiliations:** ^1^School of Finance, Qilu University of Technology (Shandong Academy of Sciences), Jinan, China; ^2^National Institute of International Strategy, Chinese Academy of Social Sciences, Beijing, China; ^3^School of International Trade and Economics, University of International Business and Economics, Beijing, China; ^4^China National Chemical Information Centre, Beijing, China

**Keywords:** economic efficiency, healthcare expenditure, BRI, DEA, environment, public health, R1, R2, R3, R4

## Abstract

Public health and the environment are the most essential pillars, and play a vital role in the economy. In order to better public health, the economic and environmental atmosphere must be stable and clean, respectively. Thus, this paper emphasizes on nexus between economic, public health, and the environment. Therefore, the objective of this paper is whether healthcare and environmental expenditures affect economic efficiency and vice versa. So, this study evaluates the performance of the country's economic efficiency and investigates the effect of healthcare and environmental expenditures for 62 Belt and Road Initiative countries for the period from 1996 to 2020. Suitable input-output variables are employed under the framework of DEA-window and Malmquist Index Productivity, and Stochastic Frontier Analysis (SFA). In addition, this study estimates the relationship between economic efficiency, healthcare, and environmental expenditures by fixed and random effects models. Therefore, the analytical outcomes reveal that countries are economically efficient. On the contrary, SFA estimation concludes that countries are found to be inefficient, because higher variation is exists in efficiency change compared to technological efficiency change and total factor productivity change on average. In addition, it is worth notable that healthcare and environmental expenditures improve the country's economic efficiency. Furthermore, public health is also influenced by economic efficiency. Thus, this study suggests that countries should better utilize given resources and invest a specific portion of national income in order to improve economic efficiency.

## Introduction

Health and environmental issues are global emergencies that transcend state boundaries. These are issues that require coordinated solutions at all levels and global engagement in order to assist countries in transitioning to a green environment and economy. To address health and environmental concerns and their severe consequences on the economy, countries are effectively engaged with health and environmental concentrations. According to a WHO report, aggregate expenditures in the healthcare sector emphasize both green economic development and establishment resilience (1, 2). Healthcare expenses are directly related to changes in economic activities, economic efficiency, and increases in demand for human health (3–5).

With rapid economic development, investment, industrialization, and urbanization particularly in those countries whose participants in the Belt and Road Initiative projects, announced by the Chinese government, healthcare expenditures are serious to be considered for analysis. Because economic activities such as investment, combustion of transportation, industrial sectors, and energy production are the primary contributors to increasing environmental pollution. Harmful particulate matter is released into the atmosphere. Consequently, a variety of anthropogenetic can increase the negative impact on public health (6, 7). Under such circumstances, the governments can direct scarce resources toward the development of public health. Additionally, authorities should assess the balance between allocating healthcare expenditures to improve people's health and economic efficiency. The fact is evident that improving people's health can contribute to economic growth, hence enhancing population wellbeing and economic efficiency. The interaction between economic activities and public health can have positive and negative effects, which may enhance the healthcare expenditures through the use of natural resources over time, particularly in BRI countries (8).

Why choose BRI countries? There are some reasons to be considered for analysis: first, BRI countries are emerging and developing economies, they are enriched with several markets such as labor market, product market, and so on. Second, they are major contributors to the global gross domestic product. Third, they are most the populous countries compared to the rest of the world, where healthcare and environmental expenditures are needed to increase at a higher level. Fourth, BRI countries are diverse in their macroeconomic management system. Fifth, these countries are responsible to produce greenhouse gases by which several diseases cause to have occurred, as a result, healthcare, and environmental expenditures tend to be increased.

This study addresses the research problems: BRI countries are much concerned about limited public budgets and fiscal sustainability. Investing in BRI's project can create some challenges for participants, e.g., debt burden and constraint budget for infrastructure development. However, economically modest countries have to face such challenges and manage their financial resources to be efficient. Despite the economic activities such as investment, public health, and environmental issues are being occurred over time. Under such circumstances, the public health and environment also could be affected in terms of expenditures. it is essential to analyze whether countries are economically efficient or not, if they are efficient, at what scale? If they are not efficient, but why. In other words, are the participant countries well-organized through using their own existing resources before initiating the projects? So, it is essential to underline the economic efficiency relationship between health and environmental expenditures.

On conversely, the majority of the studies investigating healthcare expenditures-economic growth nexus, this study explores for the ftost time (as best of our knowledge) the relationship between economic efficiency, healthcare expenditure, and environmental expenditures (9). The literature shows most cases, economic growth is determined as a re-requisite in traditional debate. None of the studies have approached the country's economic efficiency affected by healthcare and environmental expenditures in BRI's region.

This study aims to investigate the country's economic efficiency and link with public health and environment for 62 BRI's countries. It is worth mentioning that at what parameters countries are efficient, and how healthcare and environmental expenditures significantly affect the economic efficiency. Also, efficiency allows countries to attain the optimal level of economic objectives or outputs e.g., gross domestic product, quality, and quantity constraints given, or minimum level of inputs, e.g., healthcare expenditures, total labor force, capital formation, fiscal sector rating, and macro-economic management (as used in our model). Furthermore, it appears whether countries well or efficiently utilize their own existing resources over a year. This also implies that countries must manage their resources which remained properly initialized to be efficient. In addition, environmental expenditure can also improve the country's performance by investing the amount on projects related to low caron-economy, because a clean environment can provide better health that leads enhance the labor productivity.

This study motivates by making numerous contributions to the current literature; first, estimating the country's economic efficiency for 62 participant countries BRI' project. Second, investigate the relationship between economic efficiency and healthcare expenditures in the BRI's countries. Indeed, the novelty of the results sheds light on the relationship between economic efficiency and healthcare expenditures. Third, this study does not only investigate the linear impact of healthcare expenditures on economic efficiency but also examines the non-linear impact of healthcare expenditures on economic efficiency. Fourth, we also employ the non-parametric DEA-Window, which incorporates the time-varying effect based on moving average. Fifth, this research also estimates the effect of environmental expenditures on economic efficiency and healthcare expenditures. Furthermore, it concentrates the joint effect of healthcare and environmental expenditures on efficiency and vice versa.

To examine the efficiency analysis, we measure economic efficiency by applying the non-parametric and parametric approach, i.e., data envelopment analysis (DEA) and stochastic frontier analysis (SFA) over the time period 1996–2020, respectively (10). Furthermore, Malmquist index productivity (MPI) has been introduced by scales (11), decomposes efficiency into technical efficiency change, technological change, pure efficiency change, scale efficiency change, and total factor productivity change. DEA-window technique works based on principle moving average and produce efficiency over the time period of each country in the different time period (12–15). Consequently, the economic efficiency of each country in a certain period of time is different compared to its own efficiency and other countries in other periods of time. In such a way that problems associated with the DEA technique in terms of robustness can be avoided.

In the second stage, we employed an econometric approach such as dynamic panel data analysis (DPA) to formulate the partial adjustment model. Further, GMM estimate with one-and-two step (16, 17) is also implied in order to check robustness. GMM estimators do not require information about the exact distribution of the disturbances as robust. So, the disturbances are uncorrelated with instrumental variables in the equation. Also, fixed effect and random effect models are implied to capture the country-characteristic and unsystematic effects.

The paper is structured as follows. Related studies are described in the literature review' section, while the adopted methods, study scope, and data sources are discussed in the methodology section. Afterward, empirical results and their interpretation are described in the section discussion. In the end, limitations, policy recommendations, future research, and conclusions are discussed.

## Literature Review

Several studies provide evidence examination on economic efficiency, public health, and environment in multiple aspects. We describe the previous works related to economic efficiency, and expenditures concern over public health and environment with significant research contribution to the existing literature.

### Effects of Economic Efficiency

This literature strand is related to economic efficiency, previous studies measured the economic efficiency and evaluated the performance of the countries with respect to different aspects. For instance, Mustafa et al. (18) analyzed the economic efficiency of South Asian and Middle Eastern countries that participants in the BRI projects. They measured the technical efficiency by using the DEA-CCR and DEA-BCC approaches. Thus, the findings reveal that some countries e.g., UAE, India, and Middle and South Asia were found efficient on the CCR model. On the contrary, China and South Korea were found efficient on the BBC model by increasing 33% efficiency score. Interestingly, countries well-performed by increasing the outputs level with the given level of inputs, and adopted an increasing return to scale strategy. However, the study did not include the macroeconomic variables which are the most important to calculate the economic efficiency. Subsequently, De Mendonca and Nascimento (19) measured the efficiency by using the forty-two countries under the frontier approach. Findings suggest that macroeconomic variables are essential to improve efficiency and macroeconomic stability led to economic efficiency. More precisely, countries with higher development levels, inflation, and the absence of financial crisis have better performance.

In the contrast, Fernandes et al. (20) evaluated economic efficiency by using mathematical functions. Their findings denote that economic growth is the most critical macroeconomic theme, which can lead to economic efficiency and the best performance of the countries. Furthermore, countries were found efficient in terms of allocating the resources from economic growth to welfare efficiency. Zhong et al. (21) also debate that economic efficiency is an influential factor for economic development, especially energy efficiency. They used the Slack-Based Model and decomposed the efficiency analysis into pure technical and scale efficiency. Findings suggest that economic efficiency varied over time, and some units were or countries well-performed, an indication of better utilization of given resources. In the contrast, technological progress on economic efficiency was found insignificant. Yuan et al. (22) also assessed the economic and environmental economic efficiency of 61 participant countries in the Belt and Road Initiative (BRI). They argue that some countries are well-performed economically and environmentally by using convex and non-convex production methods-based non-parametric. However, some BRI countries are still underperforming either economic or environmental efficiency (23, 24).

### Healthcare Expenditures-Economic Efficiency Nexus

This literature strand is the linkage between healthcare expenditure and economic efficiency has been well-documented in earlier research. Yang et al. (25) suggested, for example, that economic output has a significant impact on healthcare spending. Economic development increases healthcare spending in the countries with the highest healthcare costs. The countries with the highest healthcare expenditures have the capacity to invest in public health in order to improve their economic situation. Similarly, Aum et al. (26) calculated that health-related activities boost economic output; less fear of COVID-19 infection, early lifting of lockdown, and working from home for fear of infection all have a significant impact on economic output in developed countries. According to Pu et al. (27), economic output has a significant impact on health expenditures in the G7 countries. Their findings show that during periods of economic prosperity (such as before the 2008 financial crisis), health expenditures increased, whereas, after the financial crisis, they decreased.

The study of Chen et al. (28) analyzed the effect of healthcare expenditures on economic output (efficiency). They debate that how much a country should spend on healthcare expenditure in comparison to other expenditures. In addition, the aggregate amount and composition of health expenditures that maximize welfare (efficient) are dependent on efficiency ideas at three levels that are frequently muddled in the argument. Macro-efficiency scores for welfare-maximizing aggregate the health spending assessment. Findings suggest that healthcare expenditures can improve economic efficiency in terms of GDP. However, poor countries still face difficulties regarding healthcare spending. Chen and Chen (29) assessed the effect of healthcare expenditures on a country's performance. The study concludes that healthcare expenditures are increased due to economic efficiency or with a rise in gross domestic product. Furthermore, healthcare expenditures have a positive impact on economic efficiency.

### Environmental Expenditures-Economic Efficiency Nexus

Some researchers have debated the dynamic link between economic efficiency and environmental expenditures, such as Can and Gozgor (30), Mehmood (31), and Zheng et al. (32). Their findings show that a higher level of economic efficiency helps to reduce harmful environmental effects. Similarly, Dogan et al. (33) contend that economic efficiency significantly mitigates environmental deterioration in OECD countries. Furthermore, Romero and Gramkow (34) discovered that economic factors have a significant impact on environmental effects. In other words, it helps to reduce greenhouse gas emissions. Following that, Boleti et al. (35) argue that economic complexity helps to improve environmental quality by reducing carbon emissions.

The studies of Ahmad et al. (36), Ahmad et al. (37), and Dogan et al. (33) contend that economic efficiency reduces environmental quality in middle-income economies while improving it in high-income economies. Following that, Neagu and Teodoru (38) investigated the relationship between economic output and the environment in the economies of the European Union. Their findings indicate that a lower level of economic output (efficiency) has a greater impact on environmental expenditures within countries. Additionally, Shahzad et al. (39) documented the negative effects of economic complexity on environmental degradation. Furthermore, other studies, such as Chu and Le (40), report an inverted u-shaped relationship between economic output and environmental expenditures. Adedoyin et al. (41) argue, however, that there is no evidence of a significant relationship between economic output and environmental degradation which increases environmental expenditures. In the contrast, Yuan et al. (22) also emphasized the environmental and economic efficiency analysis. Their findings unveil that a lower number of environmental expenditures deteriorate economic efficiency because a higher amount is required to combat environmental issues such as air pollution, as a result, economic efficiency or output may increase through multiple economic activities.

The above literature describes that most studies have been conducted in a narrow sense, i.e., carbon dioxide emissions, transport, non-economic factors, and so on. Therefore, empirical findings provide evidence for macroeconomic in terms of the contribution to the country's economic development (42–45). In contrast, our models contribute to the existing literature; first, we investigate the country's economic efficiency by using non-parametric and parametric approaches. Second, we estimate the relationship between economic efficiency and healthcare expenditures by implying econometric approaches. In addition, our model includes macroeconomic variables to be efficient, then use investment in healthcare expenditure as an external variable. Third, this research investigates the effect of environmental expenditures on economic efficiency and healthcare expenditures. Furthermore, it also estimates the joint effect of healthcare and environmental expenditures on economic efficiency and the non-linear effect of healthcare and environmental expenditures. It is worth mentioning that our mode covers the geographical area of 62 participants in BRI' projects. Recently, it has been received much global attention to analyzing the externalities of BRI projects in the countries.

## Methodology

### Study Scope and Data Source

This study focuses on 62 participants (countries) in the One-Belt One-Road project, initiated by the Chinese government year 2013 (46). Participants' countries are geographically and economically diverse and have greater potential to develop transport infrastructure. Furthermore, the countries are categorized into seven regions such as East Asia, South Asia, Southeast Asia, Central Asia, West Asia and North Africa, and Central and East Europe, including Common Wealth Independent States. Therefore, the information has been collected on suitable variants (inputs/outputs) from different international organizations namely the World Bank and OECD. In order to achieve objectives, we use data 25 years from 1996 to 2020.

Additionally, we use four inputs^1^ and two outputs i.e., the total labor force (quantity in million), gross capital formation (at current prices, million US$), fiscal sector rating (low =1, high = 6), macro-management rating (low = 1, high = 6), gross domestic product (GDP) in million US$ at current prices and human development index, respectively. Besides, we add external variables such as public-private partnership transport investment in million US$. However, Table 1 reveals the variables, measure, and source.

**Table 1:** Variable, measure, and source.

**Variable**	**Measure**	**Observer**	**Code**	**Source**
Total labor force	Number (Million)	Input	TLF	WDI
Gross capital formation	Current prices (million US$)	Input	GCF	WDI
Fiscal sector rating	Low =1, High = 6	Input	FSR	WDI
Macro-management rating	Low =1 High =6	Input	MMR	WDI
Gross domestic product	At current prices (million US$)	Output	GDP	WDI
Human development index	Score 0 to 1.0	Output	HDI	WDI
Healthcare expenditures	Percentage of GDP	Independent	HCX	WDI
Environmental expenditures	Percentage of GDP	Independent	EXP	WDI
Economic efficiency	Score 0 to 1	Dependent	EFF	Constructed

*Economic-efficiency variable is constructed by Data Envelopment Analysis (DEA) approach, and use as a dependent variable in the econometric model*.

### Theoretical Framework

**Figure 1:**
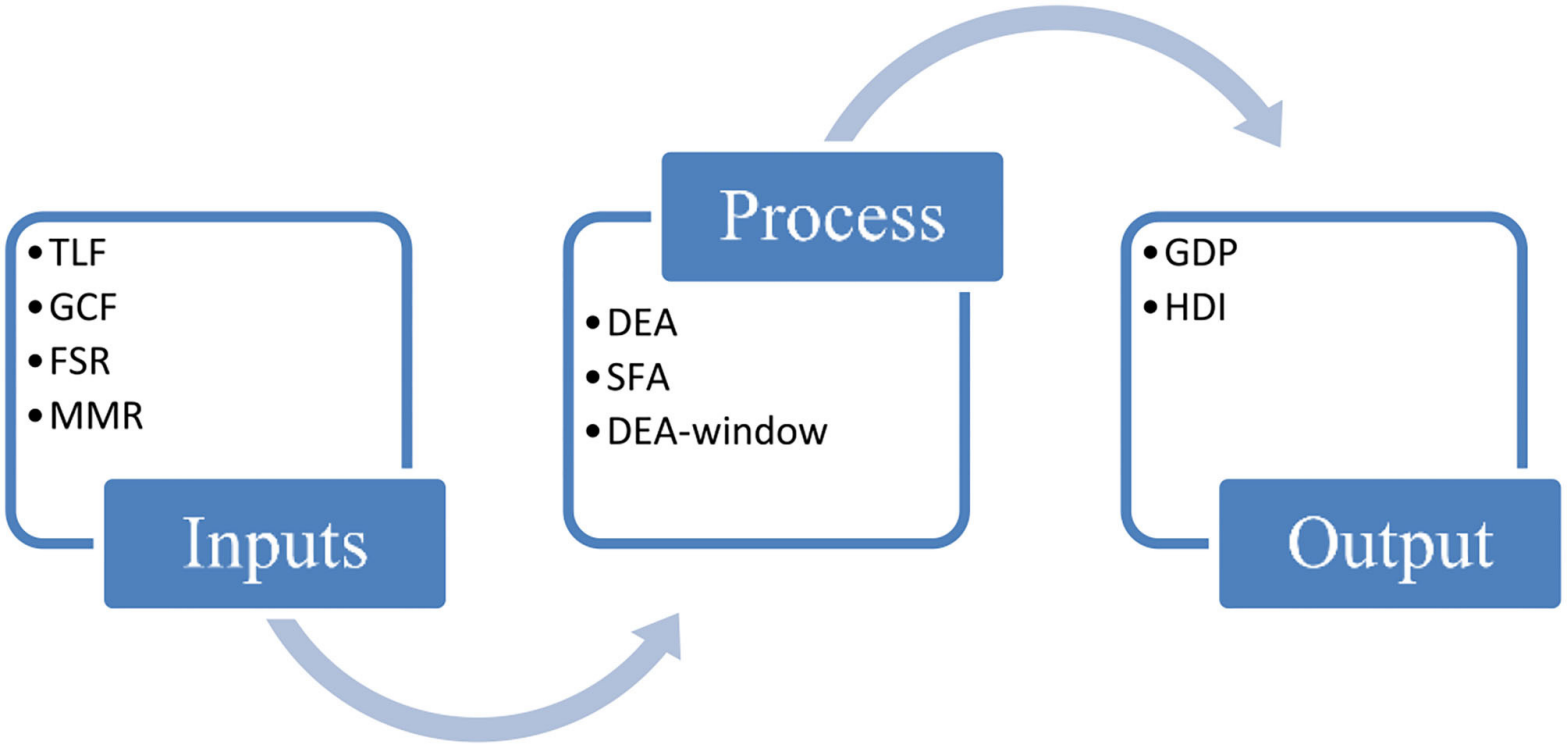
Economic transformation of resources.

Figure 1 illustrates the theoretical framework of the current model. The transformation of economic resources is occurred due to labor skills, capital, financial management.

In view of the occurrence of transformation, we shed light on inputs e.g., the total labor force is defined as all people who have the ability to work at a specific wage rate in a specific time period. The total labor force (TLF) contributes to the GDP by using education, skills, and abilities. TLF is a crucial factor for economic efficiency, because it plays a productive role in manufacturing goods and services, and adds to the GDP of an economy. Therefore, the entire input is unexplored in absence of the total labor force (47, 48).

Similarly, gross capital formation is defined as net capital accumulation within a specific time period of an economy e.g., capital goods; equipment, tools, and transportation assets. It is also a critical factor for an economy to be efficient. GCF may affect the economic-efficiency. An economy can be efficient by better utilizing the GCF, otherwise inefficient. Yasmeen et al. (49) argue that GCF has a remarkable impact on GDP, which leads to the an economy being efficient.

Subsequently, we add another variable e.g., fiscal sector rating (FSR). FSR is the most important factor for an economy. It provides a government's policy regarding the income and expenditure patterns within a time period. Beqiraj et al. (50) debate that a higher fiscal sector rating improves the economic efficiency through several channels, because fiscal policy designs the consumption patterns regarding the major projects of an economy, and while lower FSR deteriorates the economic efficiency.

Macro-management rating is also considered to be evaluated for the performance of companies, firms, and countries. Macro-management is a strategy, which stimulates the allocation of the resources in better ways so that they may produce at an optimal level. Some studies such as Anglevoa et al. (51) and Grishunin et al. (52) endorse that macro-management affect the economic efficiency, a higher level of MR may improve the efficiency score, while lower may affect EE and decline the EE score of an economy.

In addition, considering the outputs, gross domestic product (GDP) leads to economic-efficiency of an economy by increasing the goods and services. Singapai and Wu (53) argue that GDP is the best indicator of an economic-efficiency. Further, a higher level of GDP represents those economies are utilizing the better allocation of resources. Likewise, Wen et al. (54) document that an economy can be reached at an efficient level with an increase in the GDP by utilizing the given level of inputs. Lastly, the human development index (HDI) is the main output, HDI covers the life expectancy, literature rate, and gross domestic product. Zhang et al. (55) argue that economic efficiency and HDI have a significant relationship. A higher HDI value indicates that an economy is efficient, while the lower value of HDI represents the in-efficiency of an economy.

To analyze the association between economic efficiency, healthcare, and environmental expenditures, we add the healthcare and environmental expenditures in the empirical econometric model. Healthcare expenditures are defined as total expenditure on healthcare goods and services in terms of the percentage of gross domestic product. Raghupathi and Raghupathi (56) and Nathaniel et al. (57) argue that healthcare expenditures positively contribute to economic efficiency by augmenting economic growth. Environmental expenditures are defined as total expenditures on projects related to the environment in order to combat the environmental issues in terms of percentage of GDP. Hussain et al. (23) debate that environmental expenditures improve environmental quality, which could be playing a productive role in a country's performance concern over economic efficiency.

### Estimation Methods

#### Malmquist Index Productivity

Malmquist index productivity (*MPI*) calculates the change in technical efficiency over a period of time in entities or decision-making units by utilizing the multiple inputs and outputs. It can be stated as follows:


(1) $$\begin{array}{l} M_{0}\left(x^{t+1},\;y^{y+1},x^{t},y^{t}\right)=\frac{D_{0}^{t+1}\left(x^{t+1},y^{t+1}\right)}{D_{0}^{t}\left(x^{t},y^{t}\right)}\sqrt{\frac{D_{0}^{t}\left(x^{t+1},y^{t+1}\right)}{D_{0}^{t+1}\left(x^{t+1},y^{t+1}\right)}\frac{D_{0}^{t}\left(x^{t},y^{t}\right)}{D_{0}^{t+1}\left(x^{t},y^{t}\right)}} \end{array}$$

Equation (1) exhibits that the non-square root term is a change in efficiency and the square root term is called technological change produced by geometric means of two ratios over a period of times *t* and *t*+ 1.


(2) $$\begin{array}{l} \text{Efficiency change}=\;\frac{D_{0}^{t+1}\left(x^{t+1},y^{t+1}\right)}{D_{0}^{t}\left(x^{t},y^{t}\right)} \end{array}$$

(3) $$\begin{array}{l} \text{Technological change}={\left[\frac{D_{0}^{t}\left(x^{t+1}\;,\;y^{t+1}\right)}{D_{0}^{t+1}\left(x^{t+1}\;,\;y^{t+1}\right)}\frac{D_{0}^{t}\left(x^{t}\;,\;y^{t}\right)}{D_{0}^{t+1}\left(x^{t}\;,\;y^{t}\right)}\right]}^{1/2} \end{array}$$

Where *x*^*t*^,*x*^*t*+1^ are input vectors in a period of time *t* and *t*+1, while *y*^*t*^, *y*^*t*+1^ are output vectors in a period of time *t* and *t*+1. Thus, distance functions are represented in form of *D*^*t*^, *D*^*t*+1^ over a period of time *t* and *t*+1. MPI has been applied for measuring the efficiency scores of 62 countries. Furthermore, it measures the efficiency change in two periods of time (*t, t*+1) as well as the relative productivity of the countries (as in our analysis).

The possible outcomes of MPI are following:


$$\begin{array}{l} \begin{array}{l} \text{Productivity gain}=\text{MPI}>1,\text{over the time period}\left(t,\;t+1\right)\\ \text{Productivity loss}=\text{MPI}<1,\text{ over the time period}\left(t,\;t+1\right)\\ \text{No change}\equiv \text{MP I;}=0,\text{ over the time period}\left(t,\;t+1\right) \end{array} \end{array}$$

Moreover, the product of scale efficiency changes and technical efficiency change, including technical change is called productivity change.

#### Data Envelopment Analysis (Window Analysis)

The data envelopment analysis (DEA) technique measures the efficiency and deals with handling the cross-sectional and time-varying data of decision-making units (10). Further, this technique detects the efficiency trends over time by comparing decision-making units. A comparison can be possible against itself every DMU and against other DMUs over time (12). Thus, according to Tulkens and Vanden Eeckaut's (58) suggestion number of time periods is included in the analytical form. In our study, we use 62 countries (*n* = 62) for the time period from 1996 to 2000 (*T* = 25). There are no technical changes within each of the windows because all DMUs are compared (measured against each other). In order to credible results, a narrow window width must be used (12).

In our case, we choose the 3-year window analysis (*w* = 3) and then place each DMU in the window for treatment. The first window comprises 3 years (1996, 1997, and 1998) and increases DMUs from 62 to 186 (*n* × *w* = 62 × 3) for performing the analysis. While performing the analysis, the window moves on 1 year period, drops the original year and adds a new year. The procedure reaches till last window 23 containing the years 2018, 2019, and 2020 has been analyzed.

The main advantage of DEA window analysis is the provision of efficiency trends over a specified time period. Further, it evaluates simultaneous stability, properties of efficiency across and within the specified windows (14, 15, 59, 60).

#### Measuring Economic Efficiency

To measure economic efficiency, we adopt the formalization of Asmild et al. (12) and suppose that N decision-making units (*n* = 1, …*N*), T time periods (*t* = 1, …*T*), *r* and *s* are inputs and outputs, respectively. The sample size is the product of the number of decision-making units *n* and several time periods *t* i.e., (*n* × *t* = *observations*), *n* = 62, *t* = 25, *observations* = 1550. The dimensional vectors of decision-making units $\left(DMU_{t}^{n}\right)$ are following:


$$\begin{array}{l} \begin{array}{c} \text{(r) dimensional input vector and(s) dimensional output vector}\\ {\;x}_{t\;}^{n}=\;{\left(\;x_{1t\;}^{n}\;,\;x_{2t}^{n},\;x_{3t}^{n},\;\ldots \ldots .x_{1n}^{n}\right)}^{\prime },\text{and}\\ y_{t\;}^{n}=\;{\left(\;y_{1t\;}^{n}\;,\;y_{2t}^{n},\;y_{3t}^{n},\;\ldots \ldots .y_{1n}^{n}\;\right)}^{\prime } \end{array} \end{array}$$

Afterward, a window k_w_ with (*k* × *w*) observations are symbolized starting time *k* (1 ≤ *k* ≤ *T*) with width (1 ≤ *w* ≤ *T*−*k*). Thus, the matrixes of input/output are given as;

*Input's matrix*


(4) $$\begin{array}{l} x_{kw}=\left(x_{k}^{1},\;x_{k}^{2},\ldots x_{k}^{N},x_{k+1}^{1},x_{k+1}^{2},\ldots x_{k+}^{N},x_{k+w}^{1},x_{k+w}^{2},\ldots .x_{k+w}^{N}\right) \end{array}$$

*Output's matrix*


(5) $$\begin{array}{l} y_{kw}=\left(y_{k}^{1},\;y_{k}^{2},\ldots y_{k}^{N},y_{k+1}^{1},y_{k+1}^{2},\ldots y_{k+}^{N},y_{k+w}^{1},y_{k+w}^{2},\ldots .y_{k+w}^{N}\right) \end{array}$$

DEA window problem (input-oriented –constant return to scales) for decision-making units (*DMU*_*t*_) is described. This problem can be solved by following linear program:


$$\begin{array}{l} {\theta^{\prime }}_{kw\;t\;}=\min\theta ,\;⋋\theta \end{array}$$

Subject to


(6) $$\begin{array}{l} \begin{array}{c} -X_{kw}⋋+\theta {x^{\prime }}_{t}\geq 0\\ Y_{kw}⋋-{y^{\prime }}_{t}\geq 0\\ {⋋}_{n}\geq 0\left(n=1,\ldots .Nxw\right) \end{array} \end{array}$$

In order to allow for variable returns to scale (VRS), we add restriction $\overset{N}{\underset{1}{\sum }}{\;⋋}_{n}=1$ in our analysis (61), because countries have dissimilarities in economic measures and major heterogeneities. Efficiency can be affected due to the size of countries. As a result, a constant return to scale (*CRS*) is inappropriate. The best practice level of output to input varies with the size of the countries through less restrictive variable returns to scale (*VRS*) frontier.

#### Econometric Methods

Stochastic frontier analysis (*SFA*) is an econometric-based technique that estimates the source of inefficiency under specific functional forms or assumptions. This specifies the conditional mean output in comparison to common regression. A boundary or frontier has been defined by the production function, deviations from which can be observed as inefficiency. Stochastic frontier analysis (SFA) provides methods for modeling the frontier notion within a regression system such that inefficiency can be calculated. The frontier specification is following:


(7) $$\begin{array}{l} Y_{it}=\alpha_{t}+{x^{\prime }}_{it}\beta +\nu_{it}-\mu_{it}=\alpha_{it}+{x^{\prime }}_{it}\beta +\nu_{it} \end{array}$$

The Equation (7) contains output-input vectors such as yit and x_it_ for the country *i* (*i* = 62) over a period of time (*t* = 25), and vit is a random error, while u_it_ is an on sided error (u_it_ ≥ 0) in comparison to v_it_ and detecting the deficiency from the frontier ($\alpha_{it}+{x^{{\;}^{\prime }}}_{it}\beta +\nu_{it}$). We have a model in which inefficiency is quantified in differences between the countries in intercepts. However, restrictions have been placed on α_it_, as a result, several special cases were raised. In the context of pure cross-section (*t* = 1), identification requires strong assumptions regarding the distributions (*vi and ui*). But an extension and application of panel-data econometric (SFA) raised dissatisfaction with such assumptions. The inefficiency can be treated as a time-invariant country effect (in our analysis) by the first panel, α_i_ = α –u_i_. Estimates can be obtained by employing the standard panel methods. Afterward, such estimates of αi are converted into inefficiency. Literature provides explanations on the SFA regarding application in cross-section and panel-data. For cross-section, time invariance limitations can substitute for distributional assumptions. In contrast to panel-data, time in-variance assumptions are relaxed due to frontiers specification for the α_it._ Although retaining the advantages of a panel-data.

In order to examine the econometric analysis, we applied the auto-regressive distributed lag model (*AD*(*p, q*)) with dependent (p lagged times) and independent variable (q lagged times). The order of dynamics captures the direction of generalization. The likelihood-based selection criterion has been widely used for the specification of the model such as the Akaike Information Criterion (AIC) and Bayesian Information Criterion (BIC). To check endogeneity or test over-identifying restrictions, we adopt the GMM technique which is a non-linear instrumental variables estimator and based on Sargan statistics.

We reduce the general AD (1, 1) to an AD (1, 0) model checking the order of dynamics on each of the variables, omitting the insignificant dynamics, and having only the dependent variables lagged by one time and squared as regressors for transport-infrastructure investment and its squared. However, an important issue states that how a country adjusts to the long-run equilibrium level of economic efficiency. For this purpose, a partial adjustment model has been widely used.


(8) $$\begin{array}{l} \frac{\text{Eff}i_{t}}{\text{Eff}i_{t-1}}=\;\left(\frac{\text{Eff}i_{t}^{*}}{\text{Eff}i_{t-1}}\right)\gamma \end{array}$$

Equation (8) expresses that *Effi*_*t*_, *Effi*_*t*−1_ and $Effi_{t-1}^{*}$ are indicators of actual efficiency, lagged one period of actual efficiency and desired efficiency levels, respectively. While γ is the adjustment coefficient(0 < γ <1).

In our paper, we propose an econometric model to estimate the relationship between economic efficiency, healthcare, and environmental expenditures. The estimated models are given as:


(9) $$\begin{array}{r} EFFi_{it}=\beta_{0}+{\beta_{1}\;\left(EFF_{i,t-1}\right)+\beta }_{2}\left(HCX_{i,t}\right)+\beta_{3}\left({HCX^{2}}_{i,t}\right)\\ +\beta_{4}\left(EXP_{i,t}\right)+\beta_{5}\left({HCX\;*EXP}_{it,}\right)+\varepsilon_{i,t} \end{array}$$

(10) $$\begin{array}{r} HCX_{it}=\beta_{0}+{\beta_{1}\;\left(HCX_{i,t-1}\right)+\beta }_{2}\left(EFF_{it}\right)+\;\beta_{3}\left(EXP_{it}\right)\\ +{\;\beta_{4}\left({EXP^{2}}_{i,t}\right)+\beta }_{5}\left({EFF\;*EXP}_{it}\right)+\varepsilon_{it}\; \end{array}$$

Where EFF_i, t_ and EFF_i, t−1_ stand for efficiency and its lag, HCX_i, t_ stands for healthcare expenditures, HCX${\;}_{\text{i},\text{t}}^{2}$ represents the squared of healthcare expenditures, EXP_i, t_ indicates environmental expenditures, while HCX * EXP_i, t_ shows the interaction term of healthcare and environmental expenditures, *i* and *t* indicators of countries and time period, respectively, while ε_*it*_ is an error term. More precisely, the lag of economic efficiency is anticipated to have a positive or negative effect on economic efficiency. Similarly, HCX_i, t_ is projected to be a positive effect on economic efficiency, but its squared (HCX${\;}_{\text{i},\text{t}}^{2}$) is either a positive or negative effect on EFF_i, t._ In addition, EXP_i, t_ is also an important tor for economic efficiency, it is anticipated to have a positive effect on EFF_i, t_. Interestingly, the joint effect of healthcare and environmental expenditures is expected to have a positive or negative effect on economic efficiency. Subsequently, we propose a public health model (equation 9) and estimate the effect of economic efficiency and environmental expenditures on healthcare expenditures. Thus, the lag of HCX_i, t−1_ is also affecting healthcare expenditures, it is projected to have a positive effect. Likewise, the joint effect of economic efficiency and environmental expenditures (EFF * EXP) is anticipated to have a positive effect on healthcare expenditures.

Besides, the partial adjustment model is a short run function (*Y*_*t*_ = γβ_0_+γβ_1_*X*_*t*_+(1−γ)γ_*t*−1_+γ*u*_*t*_) as the current healthcare and environmental expenditures do not always be equal to its long-term level in the short run. In order to extract long-term function, we divide the short-term function by γ and reduce efficiency lagged one period.

This study expresses the hypotheses aim to investigate the effects of healthcare and environmental expenditures on economic efficiency, and whether economic efficiency and environmental expenditures affect healthcare expenditures. Thus, the current paper assesses the four hypotheses with regard aforementioned variables. For instance, the first hypothesis (H1), Is there any positive effect of healthcare expenditure on economic efficiency? The second hypothesis (H2): Is there any effect of environmental expenditures on economic efficiency? (Table 2). Third hypothesis (H3): How does economic efficiency influence healthcare expenditures? Lastly, H4: How do environmental expenditures affect healthcare expenditures?

**Table 2:** Descriptive statistics.

**Variable**	**Obs**	**Mean**	**S.D**	**Min**	**Max**
HDI	1,550	0.69	0.129	0.1	0.935
GDP	1,550	2.392	1.001	1	1.504
TLF	1,550	2.404	1.661	0.10	7.850
GCF	1,550	7.819	4.303	1	6.534
FSR	1,550	2.316	1.335	0.017	9.016
MMR	1,550	2.828	2.294	0.02	23.56
EFF	1,550	0.888	0.158	0.13	1.319
HCX	1,550	02.49	1.448	0.1	0.426
EXP	1,550	3.510	2.931	1	0.310

*Source: author's calculations.*

*All variables are converted into logarithm*.

## Empirical Results and Discussions

### Empirical Results

To examine the relationship between economic efficiency, healthcare, and environmental expenditures for 62 BRI countries, we analyze the observations of each selected variable under the central deviation framework. The outcomes reveal that observations vary within a small range over time, because of lower magnitudes of *SD* values. Moreover, the small magnitude of *SD* suggests that observations are not much scattered from the mean value. Nevertheless, the mean and *SD* values of the human development index (HDI) are 0.69 and 0.129, which implies that HDI is constricted toward central tendency. Likewise, the economic efficiency (EFF) also same practices regarding the scatteredness of the observations from the central point.

**Figure 2:**
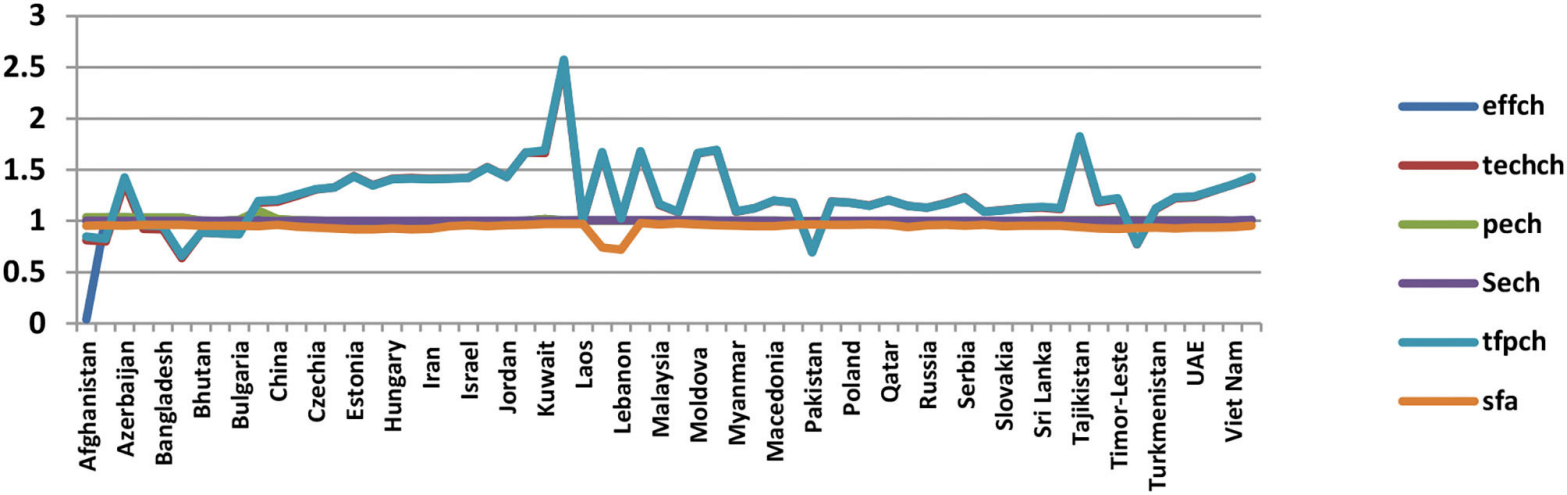
Economic efficiency analysis obtained by DEA and SFA.

Considering the measurement of economic efficiency, the outcomes from DEA and SFA approaches are reported in Table 2 and Figure 2. Results exhibit that most of the countries are technically efficient, which implies that inputs are properly utilized to achieve an optimal level of outputs. In contrast, SFA produces efficiency scores of <1 that shows the countries are neither efficient nor properly utilizing the existing resources. The reason behind that is the conversion of inputs into outputs with specific assumptions such as remaining same general price level, stable policy implication, fiscal and monetary policies, and so on. However, technical efficiency change decomposes into pure efficiency and scale efficiency. For instance, countries can reduce inputs while still remaining within the variable return to the scale's frontier (overall country well-performed by utilizing the inputs), and projected to variable return to scale efficiency frontier can further reduce their inputs (while still remaining constant return to scale).

Besides, the total factor productivity growth index decomposes into technical ones, indices of technical change, and technological efficiency change. If the technical efficiency change score is >1, this means that the best practice frontier has an increase in efficiency or catch-up impact. On the contrary, if there is <1, it indicates a decline in the country's output efficiency (62, 63).

The results provide evidence that East Asian, Southeast Asian; Central Asian countries are found to be efficient at technological efficiency changes. Further, efficiency scores are also examined in pure and scale efficiency change that provides evidence countries have substantial potential to properly utilize the resources i.e., labor force, gross capital formation, fiscal sector rating, and macro-economic management to enhance GDP and human development index (as in our analysis).

Additionally, results reveal that countries with high economic efficiency scores over the examined period are reported to be China, Mongolia, Russia, Nepal, Sri Lanka, Thailand, Vietnam, Malaysia, Central Asia, Kuwait, Lebanon, Croatia, Czech-Republic, Latvia, Lithuania, Macedonia, Ukraine, Azerbaijan, and Moldova, while only one Afghanistan is reported as most inefficient.

Stochastic frontier efficiency scores are described in the last column (Table 3). The highest inefficient countries are reported to be Georgia, Estonia, Indonesia, Sri Lanka, Iran, Hungary, Slovenia, Thailand, Egypt, Syria, Czech Republic, Tajikistan, and Timor-Leste. In contrast, countries with the lowest inefficiency scores are Latvia, Lebanon, Lithuania, Malaysia, Laos, Kyrgyzstan, Kuwait, Maldives, Pakistan, China, and Moldova. Furthermore, empirical evidence shows that countries are failing to well utilize their resources (i.e., labor force, gross capital formation, fiscal sector rating, and macro-management) around 2–8% to produce an optimal level of output (GDP).

**Table 3:** Countries' economic efficiency scores.

**Approach**	**Malmquist Index Summary**					**SFA**
**Country**	**TE**	**TECHCH**	**PECH**	**SECH**	**TFPCH**	**TE**
**East Asia**						
China	1.016	1.185	1.019	0.998	1.205	0.96
Mongolia	1.003	1.691	1	1.003	1.696	0.965
Russia	0.998	1.131	0.998	1	1.129	0.947
Mean	1.005	1.335	1.005	1.000	1.343	0.957
**South Asia**						
Afghanistan	0.039	0.814	1.039	1	0.846	0.952
Bangladesh	1.034	0.917	1.034	1	0.948	0.964
Bhutan	1	0.888	1	1	0.888	0.955
Maldives	1.004	1.086	1	1.004	1.09	0.967
Nepal	1.002	1.124	1	1.002	1.126	0.962
Pakistan	0.998	0.696	0.999	0.999	0.694	0.963
Sri Lanka	1.007	1.131	1.007	1	1.139	0.921
Mean	0.869	0.950	1.011	1.000	0.961	0.954
**Southeast Asia**						
Indonesia	0.997	1.419	0.997	1	1.414	0.92
Thailand	1.008	1.184	1.008	1	1.194	0.927
Malaysia	1.004	1.158	1	1.004	1.163	0.976
Viet Nam	1	1.354	1	1	1.354	0.95
Singapore	0.998	1.092	0.998	1	1.09	0.954
Philippines	0.999	1.19	0.999	1	1.188	0.94
Myanmar	1.003	1.09	1	1.003	1.093	0.962
Cambodia	1.015	1.179	1.1015	1	1.197	0.951
Laos	1.007	1.006	1.003	1.004	1.013	0.972
Timor-Leste	1.007	1.212	1.007	1	1.221	0.936
Mean	1.003	1.181	1.011	1.1001	1.192	0.948
**Central Asia**						
Kazakhstan	1	1.666	1	1	1.666	0.962
Uzbekistan	1.005	1.293	1.005	1	1.299	0.952
Turkmenistan	1.007	1.118	1.007	1	1.126	0.942
Kyrgyzstan	1.007	2.556	1.002	1	2.575	0.97
Tajikistan	1.008	1.813	1.008	1	1.826	0.935
Mean	1.005	1.689	1.004	1	1.698	0.952
**West Asia and North Africa**						
Saudi Arabia	0.998	1.172	0.998	1	1.169	0.953
UAE	1.006	1.231	1.006	1	1.238	0.952
Oman	0.999	1.177	1	0.999	1.176	0.965
Iran	0.997	1.41	0.997	1	1.406	0.924
Turkey	1.007	0.771	1.007	1	0.776	0.937
Israel	0.998	1.422	0.998	1	1.419	0.958
Egypt	0.999	1.33	0.999	1	1.329	0.928
Kuwait	1.018	1.661	1.018	1	1.69	0.97
Iraq	0.998	1.416	0.998	1	1.414	0.948
Qatar	0.998	1.206	0.998	1	1.204	0.955
Jordan	0.999	1.43	0.999	1	1.429	0.959
Lebanon	1.005	1.019	1	1.006	1.024	9.72
Bahrain	1.034	0.924	1.034	1	0.956	0.961
Yemen	1.009	1.418	1	1.009	1.43	0.939
Syria	1.007	1.118	1.007	1	1.126	0.93
Mean	1.004	1.247	1.003	1.000	1.252	1.533
**Central and East Europe**						
Bosnia and H	0.999	0.88	0.999	1	0.879	0.953
Bulgaria	1.009	0.879	1.009	1	0.87	0.953
Croatia	1.008	1.246	1.008	1	1.257	0.946
Czech-Repub	1.001	1.308	1.001	1	1.31	0.935
Estonia	0.999	1.438	0.999	1	1.436	0.92
Hungary	0.996	1.411	0.996	1	1.406	0.925
Italy	0.998	1.525	0.998	1	1.523	0.948
Latvia	1.006	1.662	1.002	1.004	1.672	9.74
Lithuania	1.005	1.674	1	1.005	1.681	0.979
Macedonia	1.002	1.197	1	1	1.198	0.965
Poland	0.998	1.178	0.999	1	1.176	0.957
Portugal	0.999	1.151	0.999	1	1.149	0.96
Romania	0.998	1.148	0.998	1	1.146	0.964
Serbia	0.998	1.229	0.998	1	1.227	0.953
Slovakia	0.997	1.106	0.997	1	1.103	0.94
Slovenia	0.998	1.127	0.998	1	1.125	0.927
Mean	1.004	1.247	1.003	1.000	1.252	1.533
**Common Wealth Independent States**						
Ukraine	1.007	1.224	1.007	1	1.232	0.947
Azerbaijan	1.035	1.377	1.035	1	1.425	0.952
Armenia	1.036	0.798	1.036	1	0.826	0.959
Belarus	1.034	0.637	1.034	1	0.659	0.963
Georgia	0.996	1.351	0.996	1	1.345	0.92
Moldova	1.004	1.659	1	1.004	1.665	0.962
Mean	1.018	1.174	1.018	1.000	1.192	0.950
Overall Mean	1.006	1.206	1.005	1.001	1.213	0.9513

*Source: author's calculations.*

*Inputs: total labor force, gross capital formation, financial sector rating and macroeconomic management rating. Outputs: gross domestic product and human development index. TE, Technical Efficiency; TECHCH, Technological Changes; PECH, Pure Efficiency; SECH, scale efficiency change; TFPCH, Total Factor Productivity Change; SFA, Stochastic Frontier Analysis. Economic efficiencies are obtained by non-parametric and parametric approaches such as data envelopment analysis (DEA) and stochastic frontier analysis (SFA)*.

Therefore, the economic-efficiency scores obtained by the DEA-window are reported in Table 4. The columns and rows enable us to examine the stability of econeconomic efficiency determine the trends. Thus, the stability and trend changes in economic efficiency are shown across the time periods. In comparison, China is to be an economically efficient country relative to 61 countries.

**Table 4:** DEA window analysis.

**Window/year**	**1996**	**1997**	**1998**	**1999**	**2000**	**2001**	**2002**	**2003**	**2004**	**2005**	**2006**	**2007**	**2008**
W1	0.90	0.91	0.91										
W2		0.92	0.93	0.94									
W3			0.93	0.94	0.98								
W4				0.98	0.99	1.00							
W5					1.00	1.00	1.00						
W6						1.00	1.00	1.00					
W7							0.99	1.00	1.00				
W8								0.99	0.99	1.00			
W9									1.00	1.00	1.00		
W10										0.99	1.00	1.00	
W11											1.00	1.00	1.00
W12												0.97	0.98
W13													1.00
Mean	0.90	0.92	0.92	0.95	0.99	1.00	0.99	0.99	0.99	0.99	1.00	0.99	0.99
year	2009	2010	2011	2012	2013	2014	2015	2018	2019	2020			
W12	0.99												
W13	1.00	1.00											
W14		1.00	1.00	1.00									
W15			1.00	1.00									
W16			1.00	1.00	1.00								
W17				1.00	0.99	0.99							
W18					1.00	1.00	1.00						
W19						0.99	1.00	1.00					
W20							1.00	1.00	1.00				
W21								0.99	1.00	1.00			
Mean	0.99	1.00	1.00	1.00	0.99	0.99	1.00	0.99	1.00	1.00			

*Source: author's calculation.*

*DEA window economic efficiency scores for the case of China*.

After evaluating the country's economic efficiency, we applied econometric techniques e.g., cointegration tests in order to examine the long-relationship between economic efficiency, healthcare, and environmental expenditures. The outcomes from panel unit root tests are reported in Table 5 and suggest that observed variables such as efficiency, healthcare, and environmental expenditures are stationary and non-stationary at levels and I (I). Therefore, EFF is stationary at the level and in first difference in all cases and statistically significant, while healthcare and environmental expenditures and they are squared are also found to be stationary at levels and first-difference in most cases as well statistically significant. Besides, Table 6 presents the Pedroni Co-integration tests. We reject the null hypothesis of no-cointegration at a significance level of 0.05 in eight cases. Furthermore, results indicate that a long-run relationship exists between the EFF and its influencers for the sample countries.

**Table 5:** Panel unit roots tests.

**Levels**	**Levin- Lin and Chu t**	**Breitung t-stat**	**Im. Pesaran and Shin W-stat**	**ADF chi square**	**PP-chi-square**
EFF	−18.7715	−20.4347	−20.8655	79.8985	79.8985
	(0.0000)	(0.0000)	(0.0000)	(0.0000)	(0.0000)
HCX	0.6834	22.1578	6.7779	24.6085	24.6085
	(0.7528)	(0.0000)	(1.0000)	(0.0000)	(0.0000)
HCX ^2^	3.4590	24.0857	30.6053	3.1052	3.1052
	(1.0000)	(1.0000)	(1.0000)	(0.0010)	(0.0010)
EXP	2.3451	1.4343	3.2342	4.2483	3.4321
	(0.5343)	(1.000)	(1.000)	(1.000)	(0.8732)
EXP ^2^	1.344	0.3222	1.3223	0.2123	0.2236
	(0.000)	(0.0211)	(0.0021)	(0.0000)	(0.0200)
Diff. EFF	−10.2345	−9.3483	−26.9814	194.6315	194.6315
	(0.0000)	(0.000)	(0.0000)	(0.0000)	(0.0000)
Diff. HCX	−3.2345	−3.3214	−16.1543	96.2438	96.2438
	(0.0000)	(0.000)	(0.0000)	(0.0000)	(0.0000)
Diff. HCX^2^	−4.3245	−3.2743	−14.2886	192.8121	192.8121
	(0.0000)	(0.0000)	(0.0000)	(0.0000)	(0.0000)
Diff. EXP	−1.2343	−2.3243	−4.2133	−2.0832	−1.3221
	(0.000)	(0.000)	(0.000)	(0.0002)	(0.0383)
Diff.EXP ^2^	−0.1233	−2.3443	−4.3232	−5.3344	−2.5433
	(0.0002)	(0.0020)	(0.0000)	(0.0030)	(0.0000)

*Source: author's calculations.*

*Probabilities are in brackets, Ha: Panels are stationary. TIINV is estimated by Im. Pesaran with lag 3 but insignificant. The first two lags could not estimate due to insufficient data*.

**Table 6:** Co-integration test.

	**Statistics**	**Weighted-stat**
Panel v-stat	3.8943	0.2434
	(0.0000)	(0.2345)
Panle rho-stat	−1.7643	−1.3245
	(0.0324)**	(0.0973)*
Panel PP-stat	−4.5463	−3.2143
	(0.0010)	(0.0001)
Panel ADF-stat	−3.5329	−2.4321
	(0.0000)	(0.0023)
	Stat.	Prob.
Group rho-stat	1.2345	0.7653
Group-PP-stat	−2.3436	0.0000
Group-ADF-stat	−1.7834	0.0123

*Probabilities are in parentheses.*

***p < 0.01, **p < 0.05, *p < 0.1.

In addition, in order to examine the country-specific effect, we employ the fixed and random effects models that produce different outcomes. The outcomes are provided in the Table 7 and reveal that a 1% increase in the coefficient of lag economic efficiency (EFF_t−1_) upsurges 1.1% (full sample), 94% (EA), 4% (SA), 69.1% (SE), 27% (W&NA), 77% (CA), 67% (CEE), and 79.1% (CIS) economic efficiency overtime under fixed-effect model. On the contrary, a 75% increase in EFF due to one a 1% change in EFF_t−1_ obtained by random effect. The reason behind those countries adopting previous economic strategies in different sectors of the economy is to achieve the best level of output. For instance, investing in environmental and public health projects under a macroeconomic management system, which produces the output based on previous economic efficiency. Consequently, countries are found to be efficient with different magnitudes. Other studies e.g., Singpai and Wu (53), Yuan et al. (64), and Strielkowski et al. (65) confirm our findings and suggest that the economic efficiency of lag time remarkably impacts economic efficiency.

**Table 7:** Regional analysis of BRI (economic efficiency model).

**Model/region**	**Full** **sample**	**East Asia**	**South Asia**	**Southeast** **Asia**	**West Asia** **and** ** North Africa**	**Central** **Asia**	**Central and** **East Europe**	**CIS**
**Fixed effect**	Dependent variable: efficiency							
EFF_t−1_	0.011***	0.941***	0.041***	0.691***	0.271***	0.771***	0.671***	0.791**
	(0.395)	(0.035)	(0.021)	(0.044)	(0.030)	(0.808)	(0.040)	(0.842)
HCX	1.422***	0.432***	0.752**	0.642**	0.452*	1.852*	1.321*	0.401*
	(0.401)	(0.118)	(0.685)	(0.734)	(0.837)	(0.859)	(0.421)	(0.929)
HCX ^2^	−0.393	1.323*	1.412	−6.123	−3.663	−1.233	−3.112	1.782
	(0.595)	(0.101)	(0.449)	(0.814)	(0.793)	(0.946)	(0.366)	(0.757)
EXP	0.011***	0.053***	0.493***	0.647***	0.790***	0.152***	0.134***	0.024***
	(0.420)	(0.004)	(0.013)	(0.004)	(0.008)	(0.013)	(0.003)	(0.445)
HCX*EXP	0.047***	0.008***	0.032***	0.088***	0.060***	0.004***	0.068***	0.079
	(0.023)	(0.078)	(0.151)	(0.063)	(0.115)	(0.173)	(0.034)	(0.330)
Observations	1,488	72	168	240	360	120	384	96
**Random effect**								
EFF_t−1_	0.751***	0.211***	0.361***	1.301***	0.151***	0.271***	0.291***	0.280***
	(0.673)	(0.339)	(0.211)	(0.795)	(0.406)	(0.986)	(0.352)	(0.261)
HCX	1.662***	0.652***	0.472**	0.852**	0.392***	0.652***	1.361***	0.57 1**
	(0.758 )	(0.176)	(0.284)	(0.861)	(0.272)	(0.820)	(0.292)	(0.223)
HCX ^2^	−0.213	0.123	1.522	4.463	−1.301	2.723	−3.572	5.962
	(0.974)	(0.144 )	(0.236)	(0.847)	(0.264)	(0.650)	(0.199)	(0.218)
EXP	0.330***	0.004***	0.001***	−0.020	0.001***	−0.001	8.550***	−0.001
	(0.961)	(0.004)	(0.003)	(0.002)	(0.003)	(0.002)	(0.994)	(0.685)
ECX*EXP	0.080***	0.968***	0.849***	0.895***	0.885***	0.895***	0.902***	0.827***
	(0.009)	(0.076)	(0.045)	(0.030)	(0.044)	(0.023)	(0.013)	(0.049)
Observations	1,550	75	175	250	375	125	400	100
Country	62	3	7	10	15	5	16	6

Standard errors in parentheses, ***p < 0.01, **p < 0.05, *p < 0.1.

Considering the effect of healthcare expenditures, the coefficient of HCX is positive and statistically significant for EFF in the 62 BRI countries. It implies that a 1% increase in HCX upsurges ~142% EFF by controlling the country-specific effect. On the contrary, in the full sample case, outcomes from random effect also confirm that healthcare expenditures improve the efficiency level, which implies that a 166% increase in EFF is due to a 1% change in HCX. Several studies e.g., Yang and Usman (25), Gu et al. (66), and Ahmad et al. (67) also argue that healthcare expenditures can increase economic growth which leads to economic efficiency. Furthermore, spending on health care goods and services create the income level through multiple economic channels, ultimately, investment level increases in different sector of the economy. In the contrast, the coefficient of square HCX is also found positive for all regions concerned with economic efficiency. This implies that a 1% change in HCX^2^ decreases around 39.3% in EFF under the fixed effect model, whereas, in the random effect model, a 21.3% economic efficiency decreases due to a 1% change in square of healthcare expenditures. The reason may be behind that a rise in healthcare expenditure can shorten the resources by which countries could not be achieved the best level of output (which leads to economic efficiency). Consequently, countries remained inefficient and deteriorate the efficiency level (11).

Additionally, we focus on the effect of environmental expenditures, the coefficient of EXP has a positive and statistically significant impact on economic efficiency. Environmental expenditures can increase the economic efficiency for sample countries, it implies that a 1% change in EXP increases 1.1% in EFF in the fixed-effect model. On the contrary, a 33% increase in EFF is due to a 1% positive change in EXP in the random-effect model. Precisely, the effect of environmental expenditures is relatively lower by controlling country effect is compared to random selection. Besides, the coefficient of interaction term of HCX^*^EXP has a positive impact on EFF, indicating that the joint effect of healthcare and environmental expenditures increases the economic efficiency because BRI countries require more and more public-health care goods and environmental projects in order to protect the public health and environmental quality. Furthermore, most countries are releasing greenhouse emissions into the atmosphere, which directly and directly negatively impacts public health, under such circumstances, demand for public health care goods is enhanced. Consequently, the spending level is enhanced.

We concentrate on the effect of economic efficiency and environmental expenditures on healthcare expenditures, report the outcomes in Table 8. Healthcare expenditures are also affected by previous time period, it implies that lag of HCX has a positive and statistically significant impact on current healthcare expenditures, a 1% change in HCX_t−1_ increase 1.1% under the fixed-effect model, whereas a 31.1% increase in HCX is due to a 1% change in HCX_t−1_. Furthermore, countries constraint their budgets for healthcare goods and services annually in order to maintain public health standards Consequently, healthcare expenditures are increased due to previous time periods. Besides, the coefficient of environmental expenditures also has a remarkable impact on healthcare expenditures. This implies that a 1% increase in EXP upsurges HCX by 1.1 and 31.1% under the fixed and random effect models, respectively. Other studies [e.g., (23)] argue that environmental expenditures improve environmental quality, indicating a clean environment, suggesting maintenance of the public health. Consequently, healthcare expenditures are enhanced. More interestingly, the coefficient of the square of EXP is found to be positive, which means that an increase in environmental expenditures enhances healthcare expenditure, and also a U-shaped relationship between the HCX and EXP. This implies that a 1% increase in EXP^2^ upsurges HCX ~10.1 and 33.1% in the fixed and random effect models, respectively.

**Table 8:** Regional analysis of BRI (healthcare expenditure model).

**Model/region**	**Full** **sample**	**East Asia**	**South Asia**	**Southeast** **Asia**	**West Asia** **and** **North Africa**	**Central** **Asia**	**Central and** **East Europe**	**CIS**
**Fixed effect**	Dependent variable: Healthcare expenditures							
HCX_t−1_	0.011***	0.941***	0.401***	0.691***	0.071***	0.171***	0.171***	2.791**
	(0.385)	(0.015)	(0.031)	(0.014)	(0.040)	(0.001)	(0.010)	(0.042)
EFF	0.122***	0.132***	0.252**	0.142**	0.152*	0.052*	0.021*	0.401*
	(0.201)	(0.018)	(0.605)	(0.034)	(0.037)	(0.059)	(0.021)	(0.929)
EXP	0.193	0.323*	0.412	0.123	0.663	0.233	0.112	0.782
	(0.095)	(0.111)	(0.249)	(0.014)	(0.193)	(0.246)	(0.166)	(0.857)
EXP ^2^	0.101***	0.023***	0.193***	0.047***	0.090***	0.052***	0.104***	0.014***
	(0.020)	(0.104)	(0.023)	(0.004)	(0.08)	(0.013)	(0.003)	(0.445)
EFF*EXP	0.007***	0.018***	0.052***	0.028***	0.020***	0.002***	0.038***	0.069
	(0.013)	(0.008)	(0.101)	(0.003)	(0.105)	(0.103)	(0.034)	(0.330)
Observations	1,488	72	168	240	360	120	384	96
**Random effect**								
HCX_t−1_	0.311***	0.201***	0.061***	0.001***	0.151***	0.201***	0.091***	0.120***
	(0.123)	(0.119)	(0.201)	(0.205)	(0.016)	(0.006 )	(0.012)	(0.011)
EFF	0.162***	0.652***	0.472**	0.852**	0.392***	0.652***	0.361***	0.57 1**
	(0.058 )	(0.106)	(0.084)	(00861)	(0.072)	(0.020)	(0.202)	(0.203)
EXP	1.210	0.123	0.522	0.463	1.201	0.723	1.072	0.962
	(0.974)	(0.144 )	(0.236)	(0.847)	(0.264)	(0.650)	(0.199)	(0.218)
EXP ^2^	0.330***	0.041***	0.021***	0.120	0.101***	0.012	0.500***	0.011
	(0.061)	(0.104)	(0.002)	(0.235)	(0.001)	(0.006)	(0.004)	(0.185)
EFF*EXP	0.011***	0.032***	0.043***	0.012***	0.043***	0.014***	0.002***	0.027***
	(0.009)	(0.076)	(0.045)	(0.030)	(0.044)	(0.023)	(0.013)	(0.049)
Observations	1,550	75	175	250	375	125	400	100
Country	62	3	7	10	15	5	16	6

Standard errors in parentheses, ***p < 0.01, **p < 0.05, *p < 0.1.

Considering the effect of economic efficiency, the coefficient of EFF has a positive and statistically significant impact on HCX, which implies that a 12.2% positive change in HCX is due to a 1% increase in EFF by controlling country-specific effect. On the contrary, a 1% increase in EFF upsurges HCX by 16.2% under the random effect model. Precisely, economically efficient countries can increase the healthcare expenditures, because they better utilize their given inputs or resources, and have the potential to constraint their budgets in order to maintain the public health standards. BRI countries are needed to expand healthcare expenditures, because they are facing several diseases such as typhoid, depression, anxiety, obesity, cancer, HIV aids, and so on (68–70). In addition, the coefficient of the joint effect of environmental expenditures and economic efficiency have positive effects but lower magnitudes. This means that simultaneously EFF and EXP increase the healthcare expenditures because countries can utilize their resources through a clean environment.

### Robustness and Endogeneity

This section provides estimates on GMM based first-differences and orthogonal approaches for the full sample and seven regions. It is worth notable the uncorrelation (v_it_ ) assumption is essential. Thus, asymptotically distributed tests are estimated such as first and second-order serial correlation and Sargan (71). The results in Table 9 reveal that AD (1, 0) formulation has been preferred and omits insignificant dynamics. This includes minor changes in the long-run properties. Further, variables are strictly exogenous except for lagged efficiency. Neither test suggests that the assumption of serially uncorrelated errors is inappropriate.

**Table 9:** Endogeneity.

**Model/region**	**Full** **sample**	**East Asia**	**South Asia**	**Southeast** **Asia**	**West Asia** **and** **North Africa**	**Central** **Asia**	**Central and** **East Europe**	**CIS**
**First differences**								
EFF_t−1_	0.058***	0.770***	0.117***	0.009***	0.090***	0.004***	0.097***	0.072***
	(0.028)	(0.121)	(0.0785)	(0.0661)	(0.053)	(0.094)	(0.058)	(0.487)
HCX	1.031***	1.051***	−7.871	1.461***	1.621***	5.001***	6.221***	1.651***
	(0.164)	(0.473)	(6.151)	(0.666)	(0.369)	(0.270)	(0.426)	(0.821)
HCX ^2^	−3.402	−3.082	−8.761	2.501	−2.882	1.442	−2.191	5.261
	(0.089)	(0.579)	(0.486 )	(0.654)	(0.488)	(0.215)	(0.938)	(0.657)
EXP	0.018***	0.001***	0.020***	0.001***	0.001	0.003	0.896	0.397
	(0.057)	(0.002)	(0.002)	(0.015)	(0.013)	(0.238)	(0.412)	(0.396)
ECX*EXP	0.321	0.967	3.924	0.001	0.450	0.003	0.002	0.003
	(0.021)	(0.000)	(0.344)	(0.480 )	(0.860)	(0.193 )	(0.045)	(0.320)
Sargan	233.21	69.97	158.44	230.17	343.11	115.74	365.69	232.34
Turning Point	0.152	0.171	0.102	0.292	0.281	1.733	1.42	0.156
Observations	1,426	69	161	230	345	115	368	92
**Orthogonal**								
EFF_t−1_	0.021	0.660	0.004	0.024	0.058	0.003	0.082	0.047
	(0.401)	(0.118)	(0.073)	(0.649)	(0.052)	(0.092)	(0.107)	(0.631)
HCX	−2.751	−4.901	3.711	4.381	1.371	−9.211	3.061	−2.911
	(0.495)	(0.693)	(0.441)	(0.857)	(0.921)	(0.242)	(0.958 )	(0.606)
HCX ^2^	1.452	1.312	−8.762	1.192	3.493	4.302	−2.342	5.481
	(0.327)	(0.794)	(0.486)	(0.979)	(0.917)	(0.215)	(0.290)	(0.575)
EXP	0.009	0.001	−0.001	0.007	0.013	0.002	0.006	0.004
	(0.109)	(0.002)	(0.002)	(0.002)	(0.012)	(0.002)	(0.545)	(0.224)
ECX*EXP	−1.006	0.596	3.925	−0.633	−0.450	−4.131	−0.329	−7.888
	(0.400)	(5.205)	(4.151)	(2.990)	(2.554)	(4.532)	(2.153)	(0.274)
Sargan	70.87	69.65	173.19	234.07	351.54	115.22	368.64	
Turning Point	0.945	1.868	0.212	1.838	0.196	1.071	0.653	0.265
Observations	1,488	72	168	240	360	120	384	96
country	62	3	7	10	15	5	16	6

Standard errors in parentheses, ***p < 0.01, **p < 0.05, *p < 0.1.

*Tests are conducted namely squared residual on X, Glejser test, and Harvey test*.

Besides, the inverted U-shaped relationship between economic efficiency and healthcare expenditures are estimated statistically significant coefficients. This means that the marginal rate of economic efficiency is increased, and then declined as additional healthcare expenditure is ensured. Consequently, the law of diminishing returns is shadowed. It indicates the confirmation of studies by (72) conflicting that spending and gross domestic product are substituted.

Moreover, previous studies argue that regional connectivity is also complemented for countries to be economically efficient. Expenditures and economic growth are imperative dynamics. In order to capture the heteroskedasticity effect, some diagnostic tests have been applied (i) a regression of squared residual on X, (ii) a Glejser test, and (iii) a Harvey test. All tests report that there is no evidence of the existence and significant heteroskedasticity. It is noteworthy, adjustment rate (with efficiency adjust to equilibrium values) is slow. The adjustment of economic efficiency continues around 94.2% (1–0.058) and 97.9% (1–0.021) per annum at first-difference and orthogonal. It reveals that 94.2% of discrepancies between desired and actual economic efficiency have been adjusted in a year. It also can be said that adjustment of economic efficiency has been affected within almost three periods. The reasons for slower adjustment economic efficiency must be pursued in the countries' transport policy and their advancement procedures.

In comparison, the adjustment rate of economic efficiency is estimated at around 23% (1–0.77) and 34% (1–0.66) at first-difference and orthogonal for the East Asia region, respectively. Similarly, adjustable rates are projected around 88.3% (1–0.117) and 99.6% (1–0.004) for the South Asia region in all cases. Subsequently, the Southeast Asia region challenges 99.1% (1–0.009) and 97.6% (1–0.024) adjustment rates of economic efficiency. Likewise, the regions West Asia & North Africa are challenging the adjustment rates of economic efficiency by estimating the discrepancy around 91% (1–0.09) and 94.2% (1–0.058) between actual and desired efficiency. Thereafter, such differences or inconsistency in terms of adjustable rate is also estimated at around 99.6% in all cases in Central Asia. Central and East Europe have discrepancy scores of 90.3 and 91.8% in first-differences and orthogonal. Lastly, CIS challenges 92.8 and 95.3% discrepancy between desired and actual efficiency in all cases. Therefore, the results reveal that the adjustment rate of economic efficiency substantially varied in the countries during time periods.

Moreover, turning points for the full sample are substantially different compared to sub-samples. Looking at orthogonal deviation, turning points (1.868 million US$ for East Asia and 0.212 million US$ for South Asia) suggest that East Asia need to expenditures more million US$ of investment in public health and environment compared to South Asia to be economically efficient, and South Asia also need to invest around 1.656 million US$ in healthcare goods and services to catch-up the East Asia in economic efficient perspective. Similarly, Central & East Europe needs to invest in healthcare or public health ~1.215 million US$ to catch-up East Asian economies. This explanation exhibits that investment pattern varies from region to region or country to country. For instance, East Asia.

On conversely, West Asia, CIS, and Southeast Asia including South Asia are investing lower amounts in public health, but have the potential to develop public health systems to be economically efficient. In some cases, Central and East Europe and East Asia countries invest more in public health compared to other regions, even though they have already a better public healthcare system. However, public health has a direct effect on economic efficiency through multiple healthcare networks (73, 74).

It is also worth mentioning that the hypothesis of an inverted U-shaped relationship between economic efficiency and healthcare expenditures has not been rejected. This means that healthcare expenditures remarkably impact economic efficiency.

## Conclusion and Policy Implications

This paper investigates the economic efficiency using a sample of 62 BRI countries over the time period 1996 to 2020. Countries are categorized into seven regions to explore the regional performance as well as capture the heterogeneous effects. Further, non-parametric (DEA) and parametric approaches (SFA) are applied in order to measure economic efficiency. Afterward, in the second stage, econometric analysis has been conducted.

Therefore, economic efficiency scores obtained by DEA, demonstrate that countries are overall efficient and have excess potential to better utilize the existing resources e.g., labor force, capital formation, fiscal sector, and macro-management in order to attain an optimum level of gross domestic product and human development index including healthcare and environmental expenditures. In contrast, economic efficiency scores obtained by SFA suggest that countries are inefficient to obtain an optimal level of output/economic objective at a given level of inputs (observed variables used in the model). Findings unveil that each region is found to be economically efficient except South Asia. The possible reason is a deficiency in the domestic or macro-management and fiscal sector. In addition, DEA-window suggests that China, Russia, Pakistan, Italy, UAE, Qatar, Singapore, Poland, are found to be the best efficient economic country for the BRI's project.

On conversely, both fixed and random effect models produce significant results. Also, the random effect model adopts specifies the absence of an inverted U-shaped relationship between economic efficiency, healthcare, and environmental expenditures, because of huge cross-country variation demonstrating the dynamic nature of the relationship between economic efficiency, healthcare, and environmental expenditures. In addition, healthcare and environmental expenditures impact economic efficiency by 154 and 17.6%, respectively, whereas economic efficiency and environmental expenditures impact healthcare expenditures ~14.2 and 70.15%, respectively.

However, the GMM estimate found that the turning point is well-investigated within the samples, i.e., full sample, East Asia, South Asia, Central Asia, Southeast Asia, West Asia and North Africa, Central and East Europe, and CIS. The fact that East Asia, Southeast Asia, Central Asia, and Europe regions have larger turning points in terms of contributing tcompared efficiency comparison to the full sample, South Asia, West Asia & North Africa, and CIS. It reflects that public health's planning and strategies have changed in regions and countries over the time period.

In addition, in the case of the full sample rate of adjustment are ~95% and 92% per annum implied within almost 3 years for first-difference and orthogonal, respectively. In comparison, the adjustment rates of economic efficiency are estimated around 23% and 34% for the East Asia, 88.3 and 99.6% for the South Asia, 99.1 and 97.6% the Southeast Asia, 91 and 94.2% for the West Asia and North Africa, 99.6% for the Central Asia, 90.3 and 91.8% for the Central and East Europe, and 92.8 and 95.3% for the CIS in both cases first-difference and orthogonal, respectively. The variation in the adjustment rate of economic efficiency exists due to the economy's structure and the country's economic growth. Finally, healthcare expenditure has a remarkable impact on economic efficiency for the countries and regions.

This study recommends the implications to the practitioners, health experts, economists, and policymakers based on empirical findings. Countries should examine the efficiency analysis before initiating the development projects, i.e., healthcare spending and quality development of public health whether they efficient or not, particularly previous time period (e.g., lagged year which is estimated ~98%). The macro-economic management must be improved by each country, particularly South Asian countries, West Asian and Southeast Asian to be efficient. Further, the total labor force must be better utilized or allocated in each sector of the economy. Countries must constraint the budget for public health and the environment by improving the fiscal sector. Similarly, regions must develop fiscal integration in order to expand the network (e.g., BRI) that could support investment in the long run. BRI countries should increase healthcare expenditures by ~80.7 in order to increase economic efficiency. On the contrary, BRI countries can improve economic efficiency by increasing the healthcare expenditures by around 95% through environmental. More precisely, the joint effect of public health and environmental expenditure can improve economic efficiency.

The limitations of this study are described as follows: banking and financial institutes, debt policy, energy-consumption, transport modes, trade, and political factors. The reason is that these factors have enormous cross-country variation based on domestic economic and political systems as well as geographic diversity. The efficiency scores might not be exactly investigated. In the end, this study gives directions to the researchers for future explorations. For instance, mutual agreement on trade, domestic consumption-production, modes of transport, transport-network with shared borders, land covered by the transport sector, digitalization in the transport sector, and financing would be included.

## Data Availability Statement

Publicly available datasets were analyzed in this study. This data can be found here: https://stats.oecd.org, https://www.worldbank.org/en/who-we-are.

## Author Contributions

ZH conceived the concept, analyzed, and worked on the model. CM collected the dataset, organized, and used the software. ZZ and YW worked critically and analytically on the empirical results and discussions. All authors contributed to the article and approved the submitted version.

## Conflict of Interest

The authors declare that the research was conducted in the absence of any commercial or financial relationships that could be construed as a potential conflict of interest.

## Publisher's Note

All claims expressed in this article are solely those of the authors and do not necessarily represent those of their affiliated organizations, or those of the publisher, the editors and the reviewers. Any product that may be evaluated in this article, or claim that may be made by its manufacturer, is not guaranteed or endorsed by the publisher.

## Footnotes

^1^Inputs are related to macro-economics to estimate the efficiency.
